# Differences of the midline-crossing venous drainage pattern in supraumbilical and infraumbilical regions: Angiographic study using fresh cadavers

**DOI:** 10.1371/journal.pone.0242214

**Published:** 2020-11-16

**Authors:** Seong Oh Park, Hak Chang, Nobuaki Imanishi

**Affiliations:** 1 Department of Plastic and Reconstructive Surgery, Hanyang University Medical Center, Hanyang University College of Medicine, Seoul, Korea; 2 Department of Plastic and Reconstructive Surgery, Seoul National University College of Medicine, Seoul, Korea; 3 Department of Anatomy, Keio University College of Medicine, Tokyo, Japan; University Magna Graecia of Catanzaro, ITALY

## Abstract

Current clinical and anatomical studies show that the venous problem associated with the deep inferior epigastric perforator flap results from poor midline-crossing. We examined the venous anatomy of the infraumbilical midline area and the dynamic venous flow of the deep inferior epigastric perforator flap in nine fresh cadavers. All nine abdominal specimens were harvested between the subcostal margin and the groin crease. Two specimens were used to analyze the abdominal venous anatomy, one of which was divided into two hemi-abdominal specimens. The remaining seven specimens were harvested as deep inferior epigastric perforator flaps with one major paraumbilical perforator. Venous cannulation and serial angiographic agent injection were performed in several conditions. Each specimen was radiographed using a soft X-ray system. For additional information, computed tomography (CT) angiography-visualized superficial inferior epigastric veins (SIEVs) and the supraumbilical branch were analyzed. We noted that the venous drainage between the bilateral SIEVs was easier to configure in the supraumbilical area than in the infraumbilical area. Only one to two short polygonal venous networks connect the bilateral superficial inferior epigastric veins in the supraumbilical area; however, long and multiple polygonal venous networks connect the bilateral superficial inferior epigastric veins in the infraumbilical area, which could be a predisposing factor for venous congestion. The mean distance from the umbilicus upper border to evident supraumbilical midline crossover was 18.39±4.03 mm (range: 10.10–28.49) in CT angiograms. In cadaver specimens, the mean distance was 10.87±4.85 mm (range: 4.6–18.9). Supraumbilical midline crossover was more favorable than infraumbilical midline crossover in venous flow.

## Introduction

One of the most common complications of the deep inferior epigastric perforator (DIEP) flap, which is most commonly used for breast reconstruction, is poor venous drainage. Many venous drainage issues are related to venous congestion that occurs despite patent microvascular anastomosis sites [1,2]. Venous congestion has been reported in up to 10% of DIEP flaps in a large patient series, most commonly occurring above the midline–on the contralateral side from the pedicle [3–6].

Superdrainage using the contralateral superficial inferior epigastric vein (SIEV) shows a beneficial effect in lowering partial flap loss and fat necrosis [7–10]. Meta-analysis showed superdrainage using the contralateral SIEV significantly reduces flap congestion. Although not statistically significant, a tendency of lower partial flap loss and fat necrosis was observed [11]. This suggests that the venous drainage issues associated with the DIEP flap arise from poor midline crossing; studies on the venous anatomy of the abdominal wall support this [2,3,12,13]. Hydrogen peroxide priming has shown more detailed venous anatomy and elucidated the anatomical cause of venous drainage problems of the DIEP flap [2].

However, venous structures under the umbilicus were not well visualized in previous studies, and a detailed analysis of the dynamic venous flow was not performed. In this study, we aimed to clarify the venous anatomy of the infraumbilical midline area and analyze the dynamic venous flow of the DIEP flap.

## Materials and methods

Our study of nine fresh cadavers donated to the Department of Anatomy at Keio University was approved by the Keio University School of Medicine Ethics Committee (approval no. 20070026). Written informed consent from the donor or the next of kin was obtained for the use of the human cadavers for research and education purposes. The age of the cadavers ranged from 59 to 78 years (4 men, 5 women). None of the cadavers had a history of abdominal surgery or trauma. All abdominal specimens were harvested between the subcostal margin superiorly and the groin crease inferiorly. We performed venous cannulation and serial injection of an angiographic agent in various conditions. Two specimens were used to analyze the general venous anatomy. One of them was divided into two hemi-abdominal specimens, and the other was harvested after whole-body arterial injection with lead oxide. The remaining seven specimens were harvested as DIEP flaps, which have one major paraumbilical perforator. To mimic the venous drainage pattern of the actual DIEP flap, cannulation and serial injection of a silicone rubber compound (Microfil) were performed on the main perforator artery or its vena comitantes. The injection was gently performed manually, encountering little resistance. Each specimen was radiographed using a soft X-ray system (SOFTEX, Softec, Tokyo, Japan; 5 mA, 30 kVp, 30 seconds).

We also reviewed preoperative computed tomography (CT) angiography performed from March 2019 to February 2020 for DIEP flap perforator mapping. This study was approved by the Institutional Review Board of Seoul National University Hospital (approval no. 2009-038-1155). The requirement for informed consent was waived owing to the retrospective nature of the study. Some CT angiograms showed SIEVs and their large branches in reconstruction images. Using these images, distances from the umbilicus upper border to the visible supraumbilical midline crossover branch were measured. We selected a prominent branch with a diameter was above 1 mm to ensure venous flow. Measurement was performed in cadaver specimens in the same way using Adobe Photoshop 2020 (Adobe Systems, San Jose, CA). To determine the distance difference between the CT angiogram and cadaver measurements, a Mann-Whitney U test was performed. A value of p < 0.05 was considered significant. Statistical analysis was performed using SPSS version 25 (IBM Corp., Armonk, NY, USA).

## Results

### Hemi-abdominal specimens

**Fig 1:**
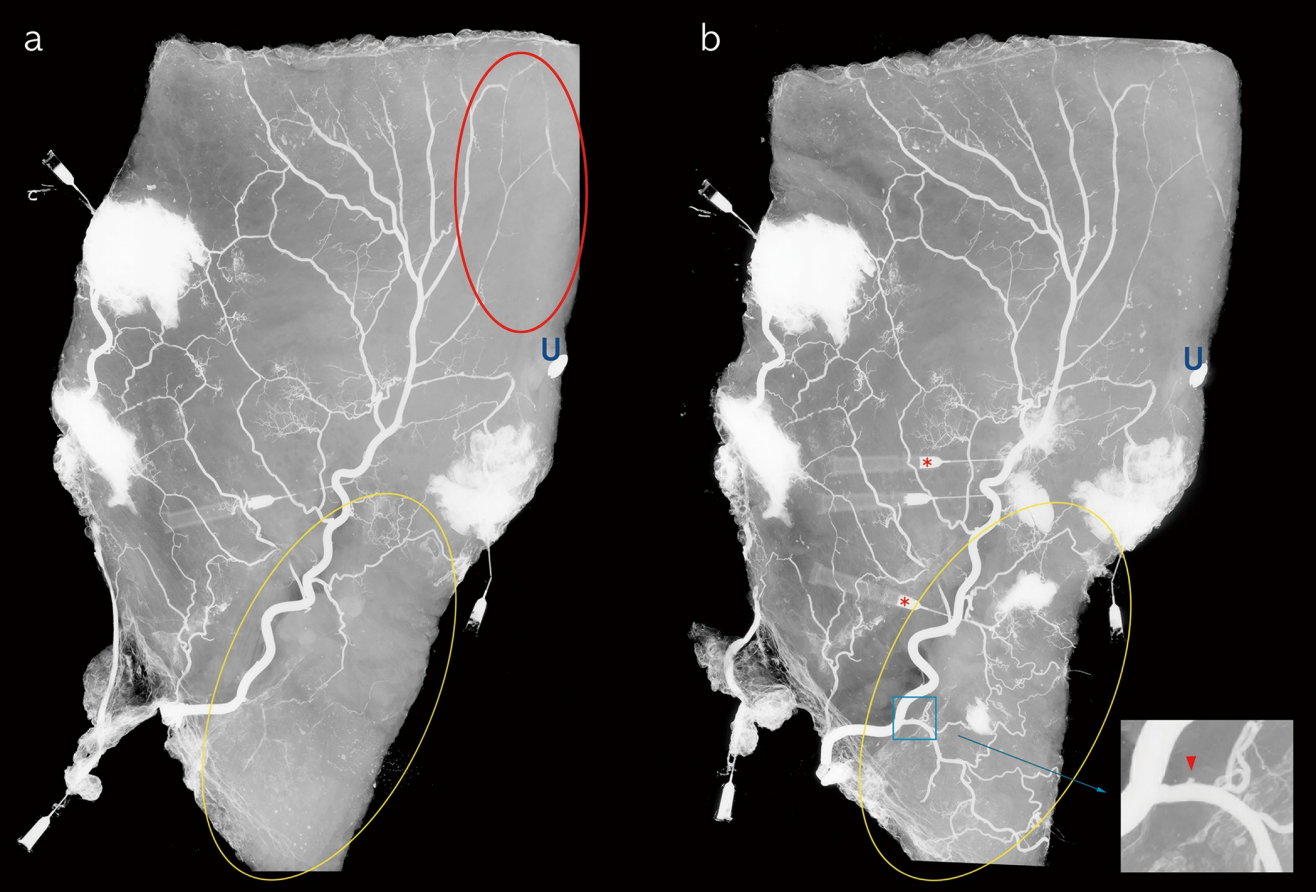
Venograms of the hemi-abdominal specimen. (a) Barium was injected through the caudal end of the superficial inferior epigastric vein and the lateral venous stump. Veins in the supraumbilical midline area were well visualized from an early period (red ellipse). (b) Advanced cannulation and injection into the medial branch of the superficial inferior epigastric vein visualized the veins in the infraumbilical midline area (red asterisks). Note the change in the infraumbilical midline area (yellow ellipse). The magnified image shows a diverticulum-like projection indicating the valve structures (red arrowhead).

**Fig 2:**
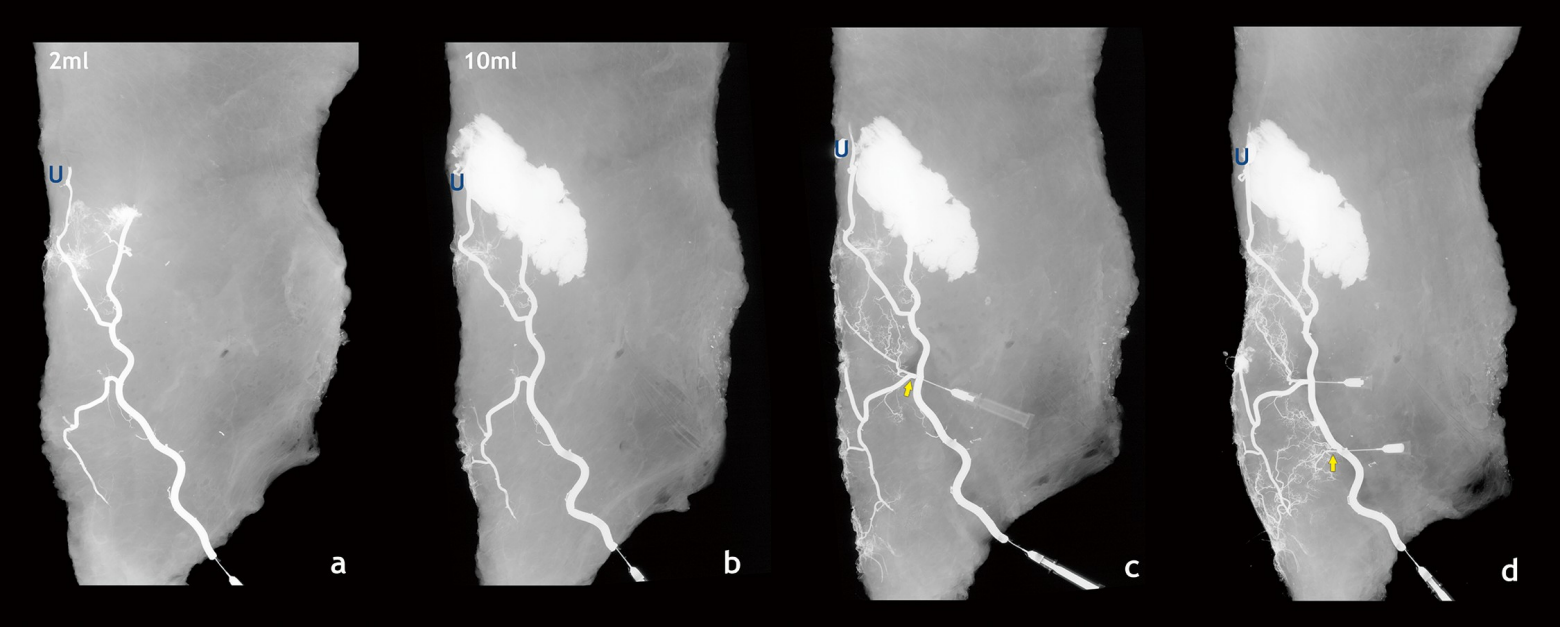
Venograms of the serial injections. (a, b) The left two venograms show serial injections (2 mL, 10 mL) through the caudal end of the superficial inferior epigastric vein. Only a part of the medial branches of the superficial inferior epigastric vein was visible despite sufficient injection. (c, d) After cannulation and injection of the medial branches of the SIEV (yellow arrow), the venous anatomy of the infraumbilical midline area was visualized, showing the fine polygonal venous network.

The caudal end of the SIEV and the lateral venous stump were cannulated, and serial injections of barium were performed to evaluate the venous pattern. Despite adequate barium injections, the infraumbilical midline area was not visualized (Fig 1A, yellow ellipse). In contrast, the supraumbilical midline area was well visualized from an early period. (Fig 1A, red ellipse) More advanced cannulation and injection into the medial branch of the SIEV allowed the veins in the infraumbilical midline area to be visualized (Fig 1B). In the other hemi-abdominal specimen, the caudal end of the SIEV was cannulated and serial injection of barium was performed to obtain venograms. The SIEV was well visualized in the cephalic direction with a small amount of barium (2 mL). Despite an additional injection, branches from the SIEV to the midline in the infraumbilical area were not visualized; instead, the barium spilled through the cephalic side (Fig 2A and 2B). Following more advanced cannulation and injection of the medial branches of the SIEV, a fine polygonal venous network was clearly shown (Fig 2C and 2D). This suggests that even if a sufficient amount of angiographic agent is injected, it may not proceed to the infraumbilical midline. We also observed that there were diverticulum-like projections, i.e., valve structures [14], which interfered with venous flow from the SIEV to the infraumbilical midline. (Fig 1B) Therefore, venous flow from SIEV to the infraumbilical midline is not favorable as it can be interrupted by valve structures.

### Arteriogram and venogram

**Fig 3:**
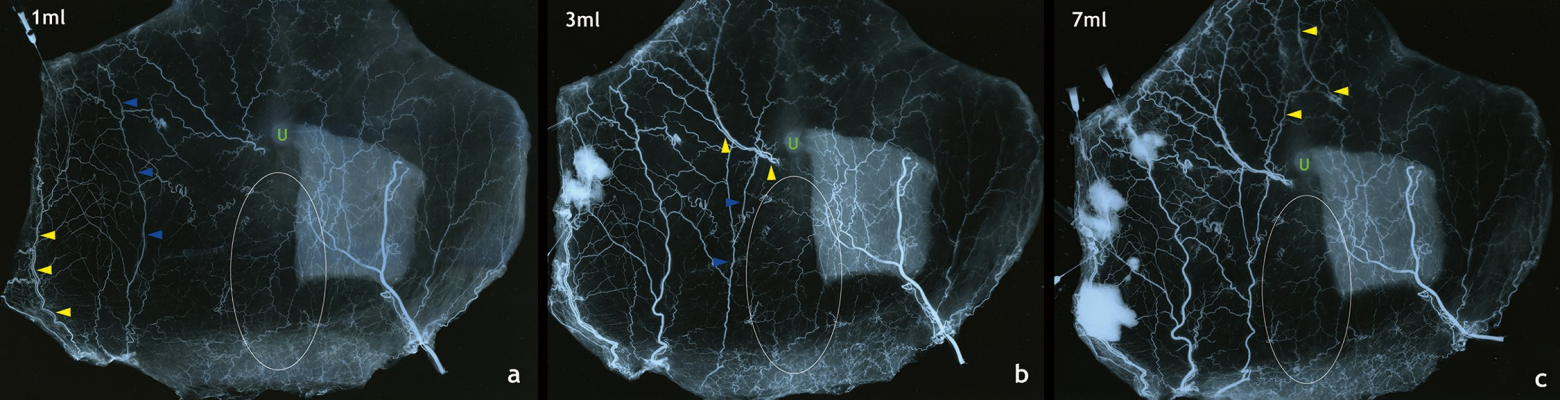
Serial venogram through the upper lateral venous stump after pre-injection of lead oxide into the arterial system. (a) The venogram taken after 1 mL injection of barium shows the vena comitantes of the superficial circumflex iliac artery (yellow arrowhead) and the superficial inferior epigastric artery (blue arrowhead). (b) The venogram taken after 3 mL injection of barium shows the medial main superficial inferior epigastric vein (blue arrowhead) and the vena comitantes of the periumbilical major perforator (yellow arrowhead). (c) The venogram taken after 7 mL injection of barium shows midline crossover in the supraumbilical area (yellow arrowhead). Note that there is no visible change in the infraumbilical midline area (white ellipse).

To confirm the relationship of the vein with the artery, lead oxide was injected into the arterial system of a cadaver. Subsequently, a transverse rectus abdominis musculocutaneous flap was harvested with the pedicle as the left deep inferior epigastric artery. One of the upper lateral venous stumps was cannulated, and barium was injected serially. When 1 mL of barium was injected, the vena comitantes of the superficial circumflex iliac artery and superficial inferior epigastric artery were visualized first. When 3 mL was injected, the barium reached the medial main SIEV and the vena comitantes of the periumbilical major perforator. When 7 mL was injected, the vein that crosses the midline in the supraumbilical area was visualized. However, there was no visible change in the infraumbilical area (Fig 3). No significant change was observed when 10 mL was injected. With these venograms, we could determine that the venous flow (midline-crossing of venous flow) started in the supraumbilical area and was difficult in the infraumbilical area. This was consistent with the venograms of the hemi-abdomens.

### Venogram through the DIEP and vena comitantes

Serial injection of Microfil into an artery or vena comitantes of the major perforator of the deep inferior epigastric artery was performed in seven fresh cadavers to evaluate the physiologic flow of the DIEP flap. When the perforator artery was injected, the ipsilateral SIEV was visualized first, followed by the midline-crossing vein in the supraumbilical area and the contralateral SIEV. However, midline-crossing veins (or the medial branch of SIEV) in the infraumbilical area were visualized very weakly. We tried to evaluate the physiologic flow through injection of the perforator artery; however, the angiographic images were unclear due to the relatively long course of the contrast medium.

**Fig 4:**
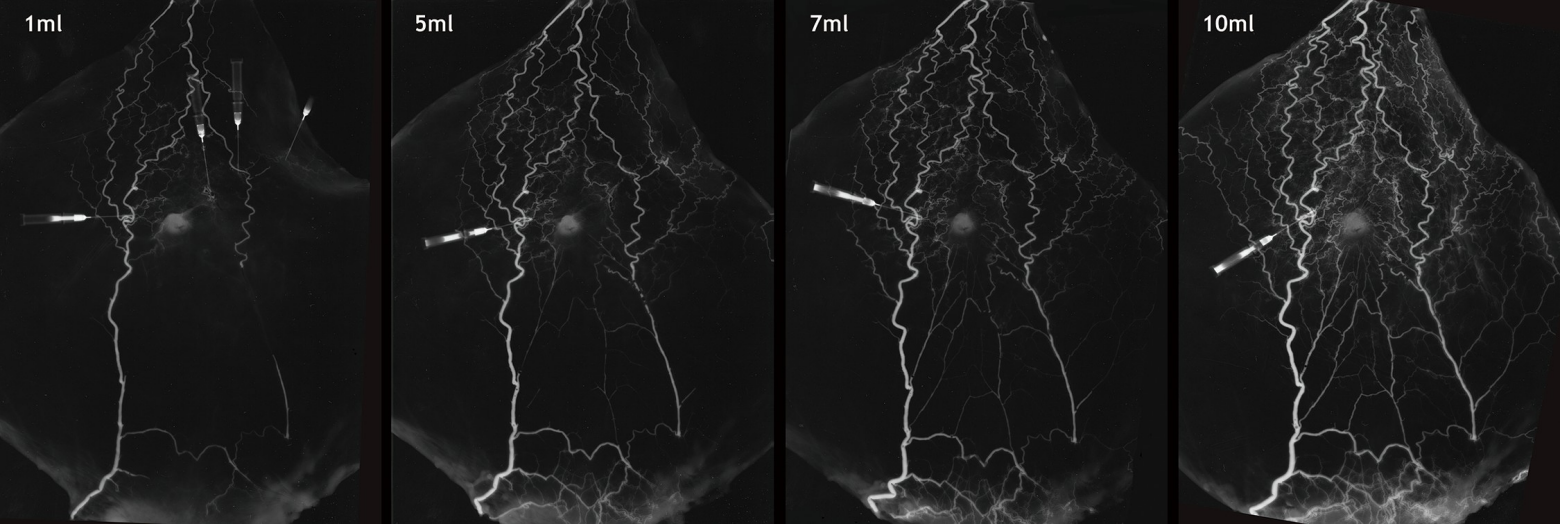
Serial venogram through the vena comitantes of the major perforator of the deep inferior epigastric artery. The venogram taken after 1 mL injection of barium shows midline crossover in the supraumbilical area. However, midline crossover began to gradually appear in the infraumbilical area with increases in the amount of injected barium.

Serial injection to the vena comitantes of the major perforator showed a direct relation among the venous networks. During serial injections of 1, 5, 7, and 10 mL of barium, the ipsilateral SIEV and the supraumbilical midline-crossing veins were visualized first. Subsequently, the contralateral SIEV was visualized. The midline-crossing vein in the infraumbilical area began to appear faintly with the 3 mL injection and became clear with the 10 mL injection (Fig 4).

### Summary of results

**Fig 5:**
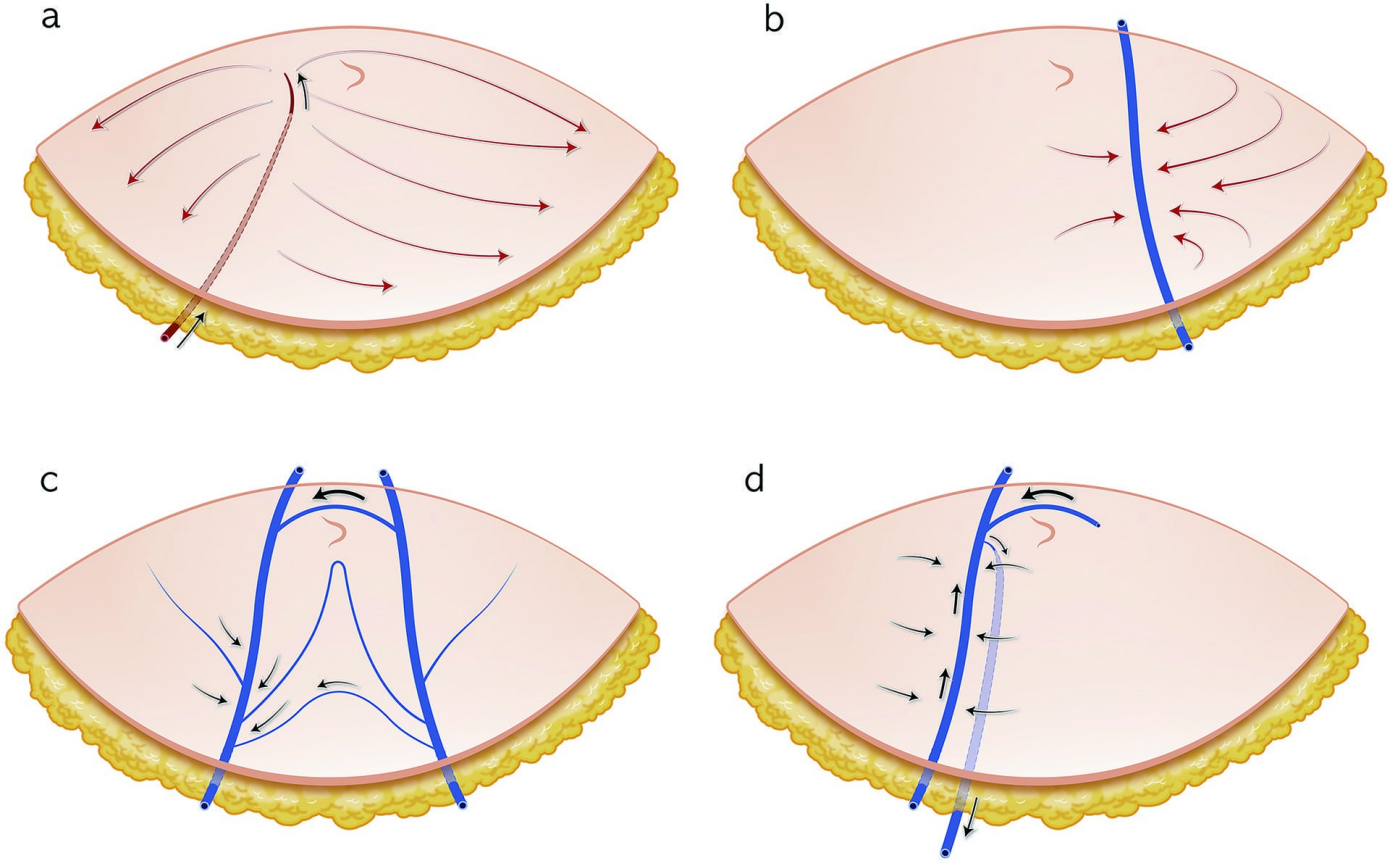
Schematic illustration of the blood flow of the entire deep inferior epigastric perforator flap. (a) Arterial inflow proceeds through the perforator artery. (b) Contralateral venous flow proceeds mainly through the superficial inferior epigastric vein. (c) Midline crossing proceeds through the supraumbilical and infraumbilical midline and gathers in the ipsilateral superficial inferior epigastric vein. (d) Venous outflow proceeds through the vena comitantes of the perforator artery.

**Fig 6:**
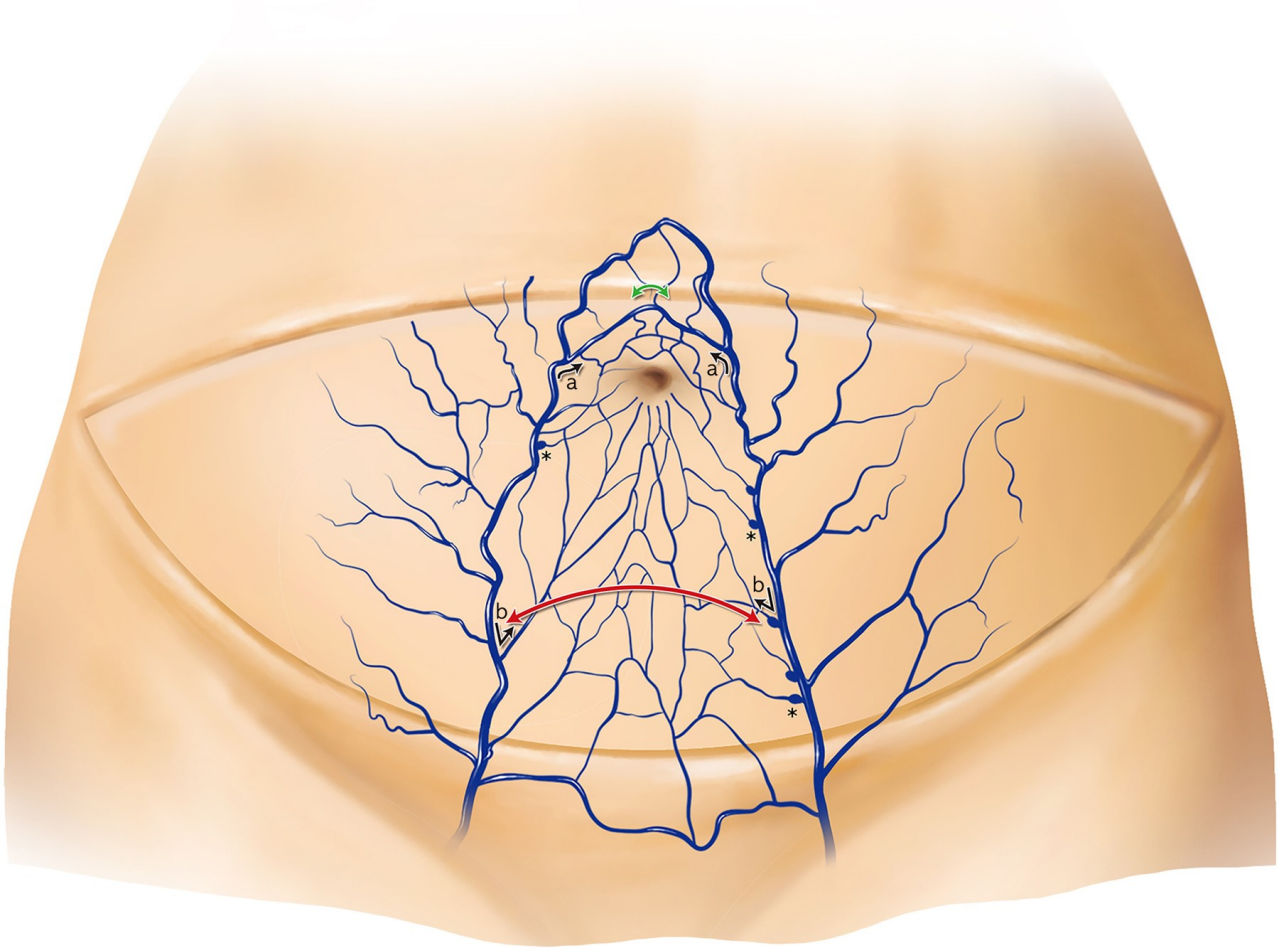
Schematic illustration of the venous structure of the lower abdomen. The supraumbilical midline crossover shows a shorter distance than the infraumbilical midline crossover (green curved arrow vs. red curved arrow). From the main superficial inferior epigastric vein to the medial branch, the angle is more acute in the infraumbilical area (b) than in the supraumbilical area (a) according to the venous flow in the deep inferior epigastric perforator flap. Diverticulum-like projections: valve structures are present near the point of contact of the medial branches of the superficial inferior epigastric vein (asterisks).

Our angiographic findings can be summarized as follows:

1. The venous drainage between the bilateral SIEVs was easier to configure in the supraumbilical area than in the infraumbilical area. Bilateral SIEVs were shaped like an inverted-V shape. The distance between the bilateral SIEVs became shorter upward and longer downward. One to two short polygonal venous networks connect the bilateral SIEVs in the supraumbilical area; however, long and multiple polygonal venous networks connect the bilateral SIEVs in the infraumbilical area. This long and complex venous course could be a predisposing factor for venous congestion.
2. The direction of venous flow from the main SIEV toward the midline below the umbilicus is unfavorable. This could be deduced from three backgrounds. First, the existence of valves at the beginning of the medial branch from the main SIEV; the direction of drainage is from the branch to the main SIEV. Only after additional cannulation above this structure were the fine venous networks in the infraumbilical area shown. Second, the angulation of the SIEV with the infraumbilical branch according to venous flow in the DIEP flap was notable. The flow from the infraumbilical medial branch to the SIEV was favorable with easy drainage. However, the flow from the SIEV to the infraumbilical branch was unfavorable. Third, the flow to the main SIEV was mostly transferred to the contralateral SIEV through midline crossing in the supraumbilical area, as shown by the serial injections (Figs 5 and 6).

### Measurement of supraumbilical midline crossover

**Fig 7:**
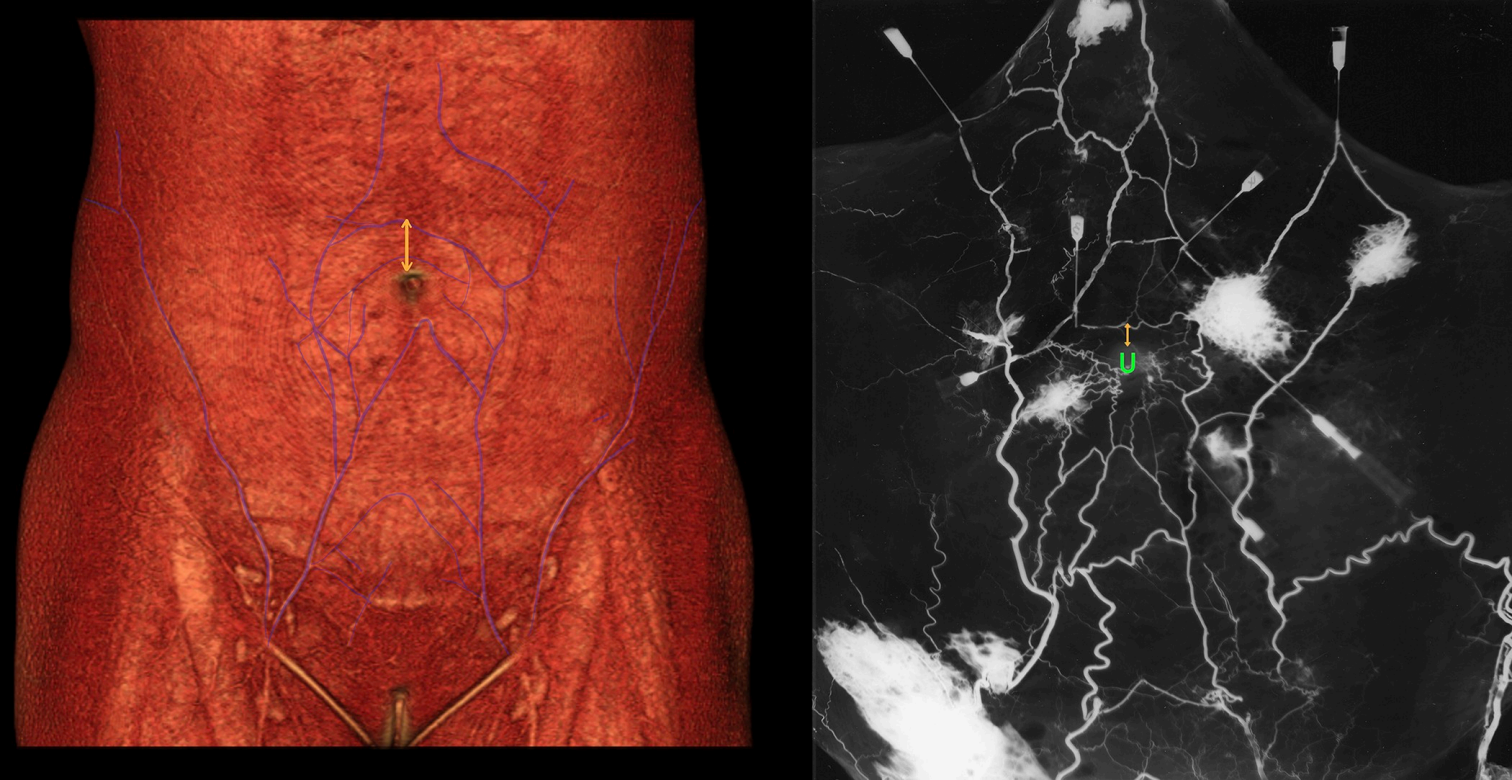
Example of measurement from the upper border of the umbilicus and the visible (>1 mm) supraumbilical midline crossover. (Left) From a preoperative computed tomographic angiogram with reconstruction. Note that the closest supraumbilical midline crossover is skipped. (Right) From an angiographic study using cadavers.

**Fig 8:**
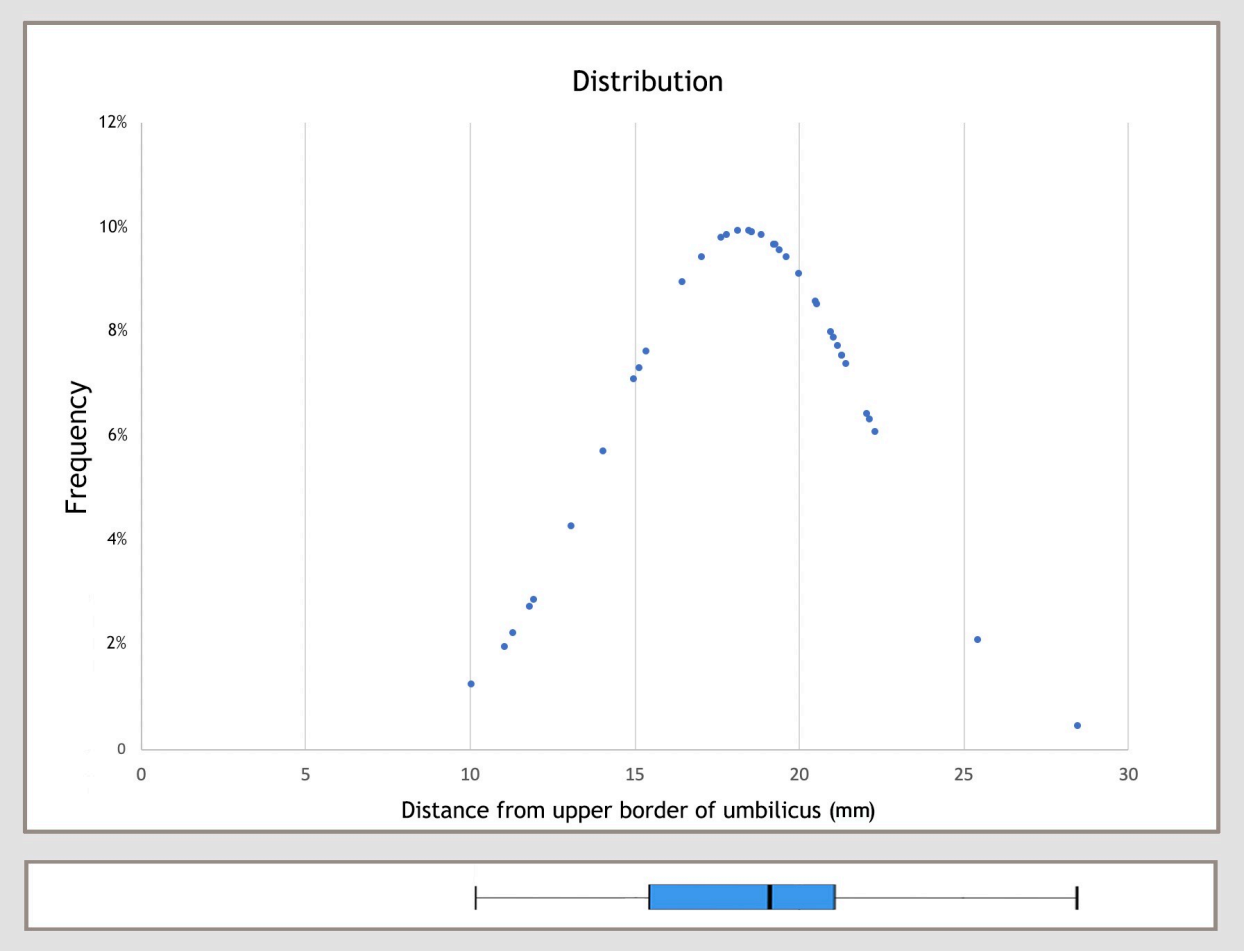
Distribution curve showing the distance between the upper border of the umbilicus and the visible supraumbilical midline crossover.

A total of 38 of 69 CT angiograms were analyzed (Fig 7). The mean distance from the umbilicus upper border to the supraumbilical midline crossover was 18.39±4.03 mm (range: 10.10–28.49 mm). In cadaver specimens, the mean distance was 10.87±4.85 mm (range: 4.6–18.9 mm). In Mann-Whitney U test, the difference between the CT angiograms and cadaver specimens was significant (p<0.001). This difference comes from CT angiogram showing only the relatively large and partial branches of SIEVs (Table 1 and Fig 8).

**Table 1:** Mean distance from the umbilicus upper border to the supraumbilical midline crossover.

	N	Mean	SD	Min	Max	P
CT angiogram	38	18.39	4.03	10.1	28.49	< 0.001*
Cadaver	7	10.87	4.85	4.6	18.9	

* Mann-Whitney U test. SD, standard deviation; Min, minimum; Max, maximum; CT, computed tomography.

## Discussion

Because of the relatively frequent occurrence of venous drainage complications associated with the DIEP flap, there have been several trials to improve the technique. The benefit of venous superdrainage using the contralateral SIEV has been demonstrated in several clinical studies [7–11]. Anatomical studies have also been conducted to understand the mechanism of the venous drainage, especially that concerning the midline crossover.

Blondeel et al. injected Microfil into the SIEV and observed a large midline-crossing vein in 18% of specimens, a smaller venous network in 45% of specimens, and no medial cross-linking branch in 36% of specimens [3]. Schaverien et al. used dynamic CT venograms to show that the bilateral SIEVs were connected by a midline-crossing vein at the subdermal plexus level. There were several venous morphologic patterns, but in some patients, no midline-crossing vein was identified [15]. A cadaveric study by Rozen et al. observed the midline crossover near the arcuate line in 15 out of 16 specimens; in some specimens, additional midline-crossing veins were observed immediately infraumbilically and supraumbilically. However, in CT venograms, the midline crossover was observed under the arcuate line in 86% of cases, and in some cases, additional midline crossover was observed near the umbilicus [13].

Despite these efforts, details of the venous anatomy of the abdomen have not been clarified in comparison with other parts of body due to the technical difficulty associated with such research. Moreover, retrograde perfusion is ineffective in this area because of the presence of valves, such as in the extremities. This difficulty can be overcome in the extremities by anterograde perfusion using a tourniquet [13]; however, this is impossible in the abdomen. Lie et al. weakened the valves using hydrogen peroxide priming and showed the venous anatomy of the abdomen clearly by venogram [2]; the midline crossover of the SIEV was shown in both the supraumbilical and infraumbilical areas. The infraumbilical venous crossover was connected to the main SIEV near the suprapubic crease. They suggested that at least one of these (the supraumbilical crossover or the suprapubic crease) should be included in the flap for better venous drainage. The hydrogen peroxide priming method enabled better visualization of the venous structure than that in previous studies; however, this method has shortcoming since the weakened valve structures are not a physiological condition.

We adopted the study method that used a serial angiographic agent injection and soft tissue radiographic system. We tried to overcome the interferences from the valve structures through additional cannulation of fine venous structures by maintaining physiological conditions. The presence of valves in the infraumbilical area is unfavorable to the DIEP flap because the valves disturb the midline crossover of venous flow. However, this condition is favorable under the normal physiological condition because it results in a better downward physiological flow for drainage. Therefore, although performing an angiographic study with maintained valve structures is more difficult, it can show a more physiological venous flow, as observed in this study. This method can be considered a conventional method; however, the soft tissue radiographs have the advantage of visualizing fine vascular structures, such as small-caliber vein, artery, and valve structures [14,16].

Through these fine visualizations, we found several meaningful results. First, the existence of valves around the root of the medial branch of the SIEV in the infraumbilical area was confirmed, and clear visualization of the fine venous network in the infraumbilical region was possible through a technically sophisticated cannulation and injection of a contrast medium. Further, we demonstrated that the midline crossover in the supraumbilical area is simpler than that in the infraumbilical area, based on structural differences. We also confirmed through serial injection that the midline crossover in the supraumbilical area preceded that in the infraumbilical area.

A goal of this anatomical study was to obtain findings that will bring clinical improvement. We applied the suggestions of Lie et al. during the elevation of the DIEP flap, including both the periumbilical perforator and the adjacent area to the suprapubic crease [2]. However, the flap was too large to adequately close the donor site. Lowering the flap could be helpful for closing the donor site. However, clinical results of low DIEP flap are not favorable; low DIEP flaps show a relatively high rate of venous congestion [17]. Although not all conditions matched perfectly in that clinical study, inclusion of only the suprapubic area could not solve the venous drainage problem. This is consistent with our results, which suggest that midline crossover is unfavorable in the infraumbilical area.

Only some of the CT angiograms used in the study showed venous structures clearly; not all branches were seen finely in contrast to anatomic study. Therefore, we selected a supraumbilical branch that had a relatively large diameter to achieve reliable information. We also selected a branch slightly away from the umbilicus; branches adjacent to the umbilicus could be damaged during the incision and dissection around the umbilicus. We recommend raising 2.5 cm of the flap upper margin to include a part of the supraumbilical midline crossover. Including the entire midline-crossing vein is impossible, because it is located too high. Instead, including only a part of the supraumbilical midline-crossover could be helpful for venous drainage; as shown by our results, the supraumbilical midline-crossover is more favorable. It is not recommended to include the subcutaneous fat tissue through beveling, instead of through an upper skin incision, because the SIEV is just below the dermal level in this region. It is difficult to raise the flap upper margin because it could induce an upper, visible scar. However, in specific clinical conditions, it could be applicable. The flap upper margin is often designed to reach the umbilicus or above in patients with relatively less redundant abdominal tissue. If there is a need for a high inset rate [18], this could be helpful because relatively large amounts of tissue above the midline would be included to the insetted flap. Raising 2.5 cm of the flap upper margin could allow for more efficient surgery and economical flap usage without an additional superdrainage procedure.

Our study had several limitations. First, the number of cadavers was small. Therefore, it was impossible to identify an important anatomical landmark and measurement that reached statistical significance. We could show only the tendencies of the macroscopic view of the venous structure. Moreover, the specimens were whole abdominal specimens that were not in the same condition as the DIEP flap used for breast reconstruction. The whole-abdominal specimens showed that the midline crossover in the supraumbilical area was more favorable. However, in this study, we could not analyze any differences in the mechanism of venous drainage when the DIEP flap is elevated in the clinical situation; In most clinical conditions, midline crossover in the supraumbilical area is impossible after DIEP flap elevation. This is only a hypothesis that was inferred from this anatomic study, especially about raising the flap upper border for capturing the supraumbilical midline crossover. This should be further validated in a follow-up clinical study.

## Conclusion

This cadaveric study showed that the supraumbilical midline crossover was more favorable than infraumbilical midline crossover in various injection conditions. This was because of the overall configuration of the SIEV (inverted-V shape) and the detailed differences in anatomical features between the SIEV and the midline-crossing branch of the SIEV in the supraumbilical and infraumbilical area, i.e., the valves, angulation, and major flow.

## Supporting information

S1 File High resolution. (ZIP)
