# Supplementary figures and images for: Differences of the midline-crossing venous drainage pattern in supraumbilical and infraumbilical regions: Angiographic study using fresh cadavers
Source: PLoS One. 2020 Nov 16;15(11):e0242214. doi: 10.1371/journal.pone.0242214 (PMC7668592; doi:10.1371/journal.pone.0242214)

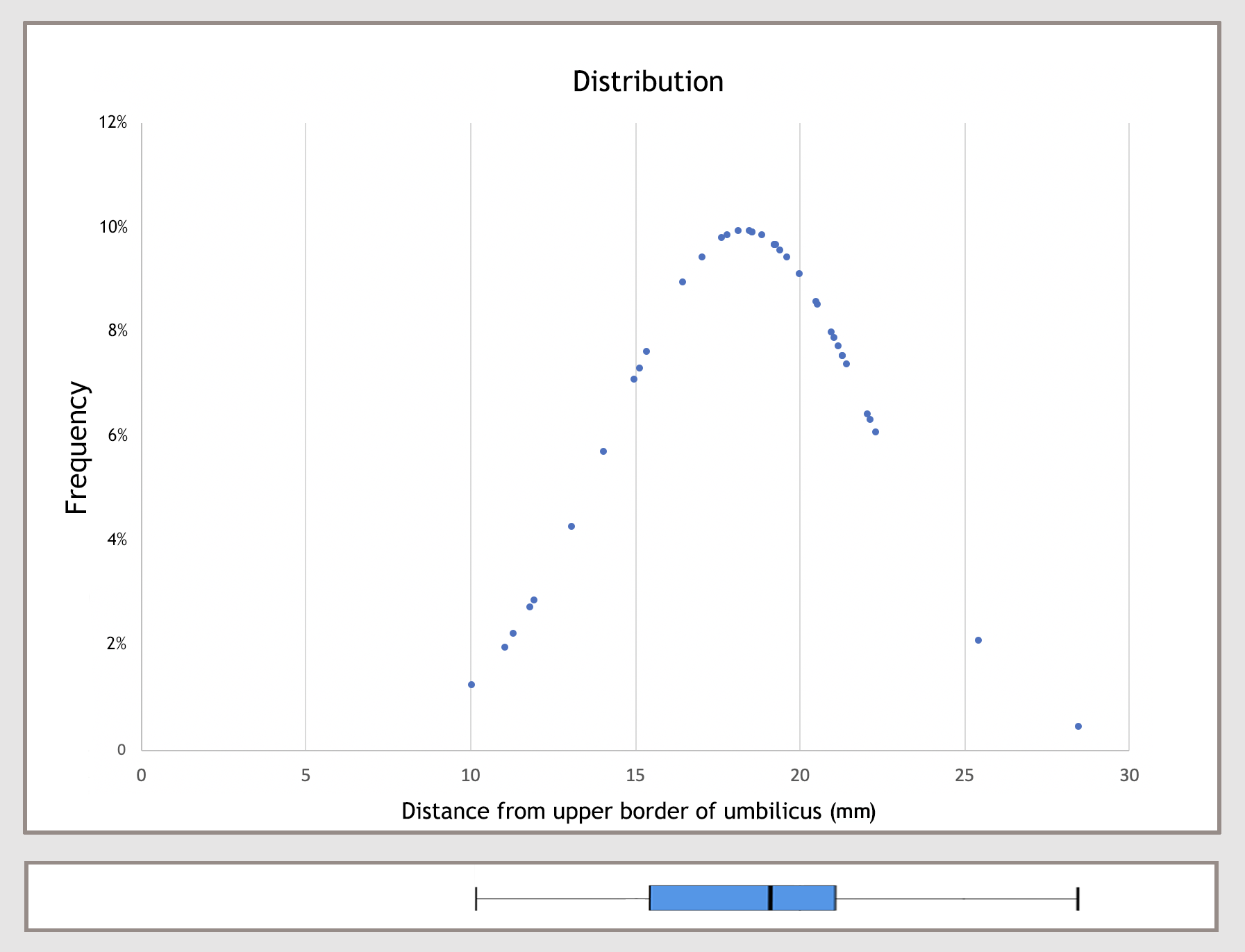

Supplement: S1 File — (ZIP) [file pone.0242214.s001.zip › Figure8.tif]

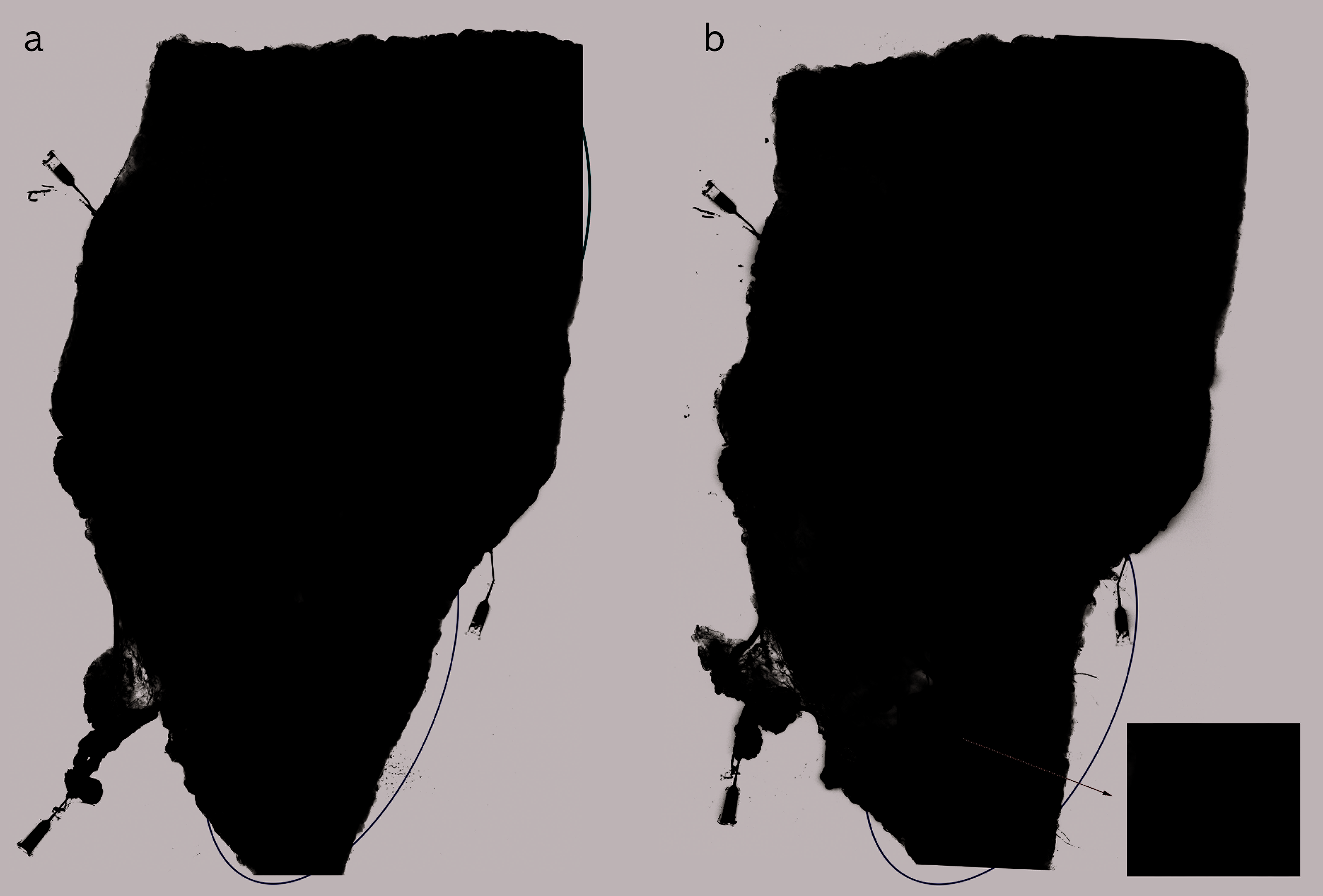

Supplement: S1 File — (ZIP) [file pone.0242214.s001.zip › Figure1.tif]

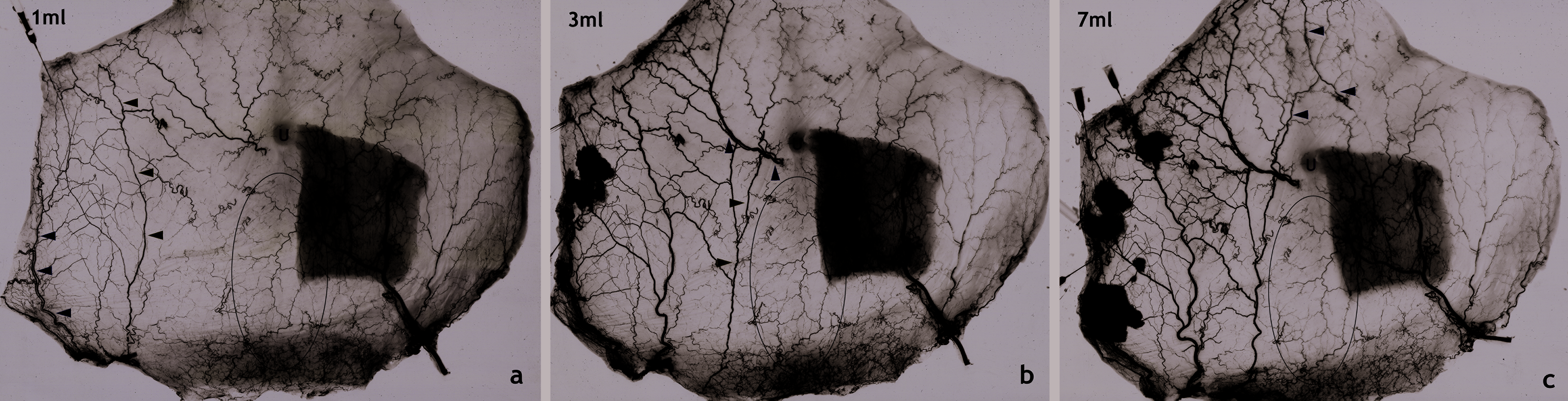

Supplement: S1 File — (ZIP) [file pone.0242214.s001.zip › Figure3.tif]

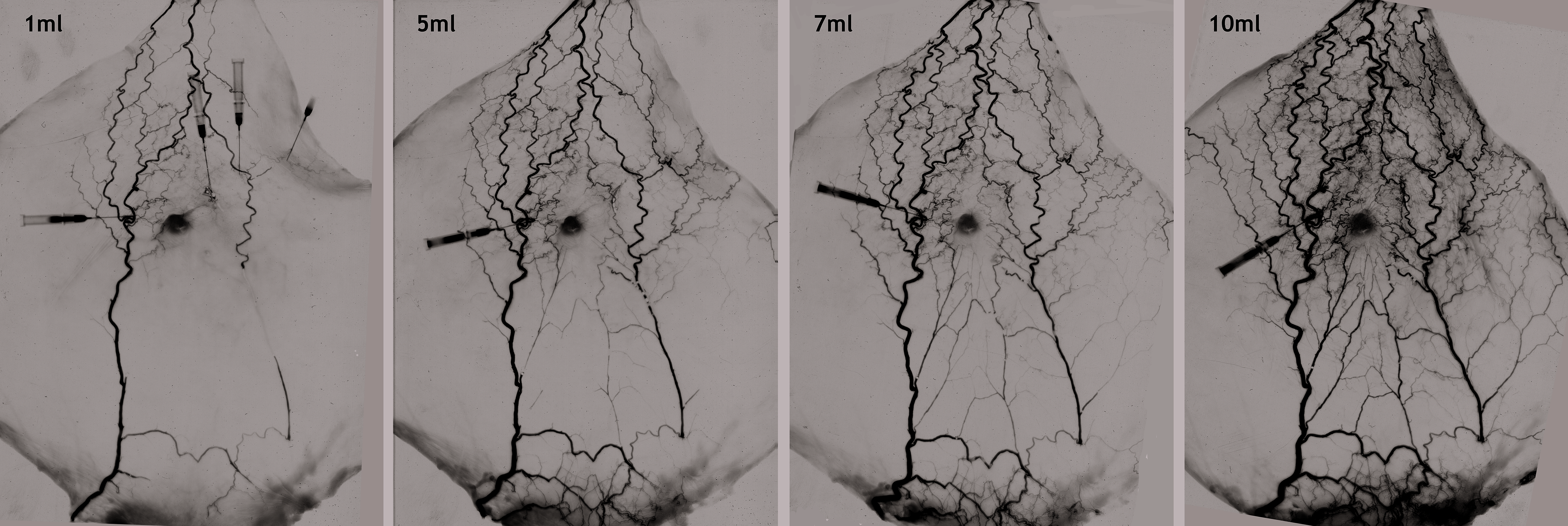

Supplement: S1 File — (ZIP) [file pone.0242214.s001.zip › Figure4.tif]

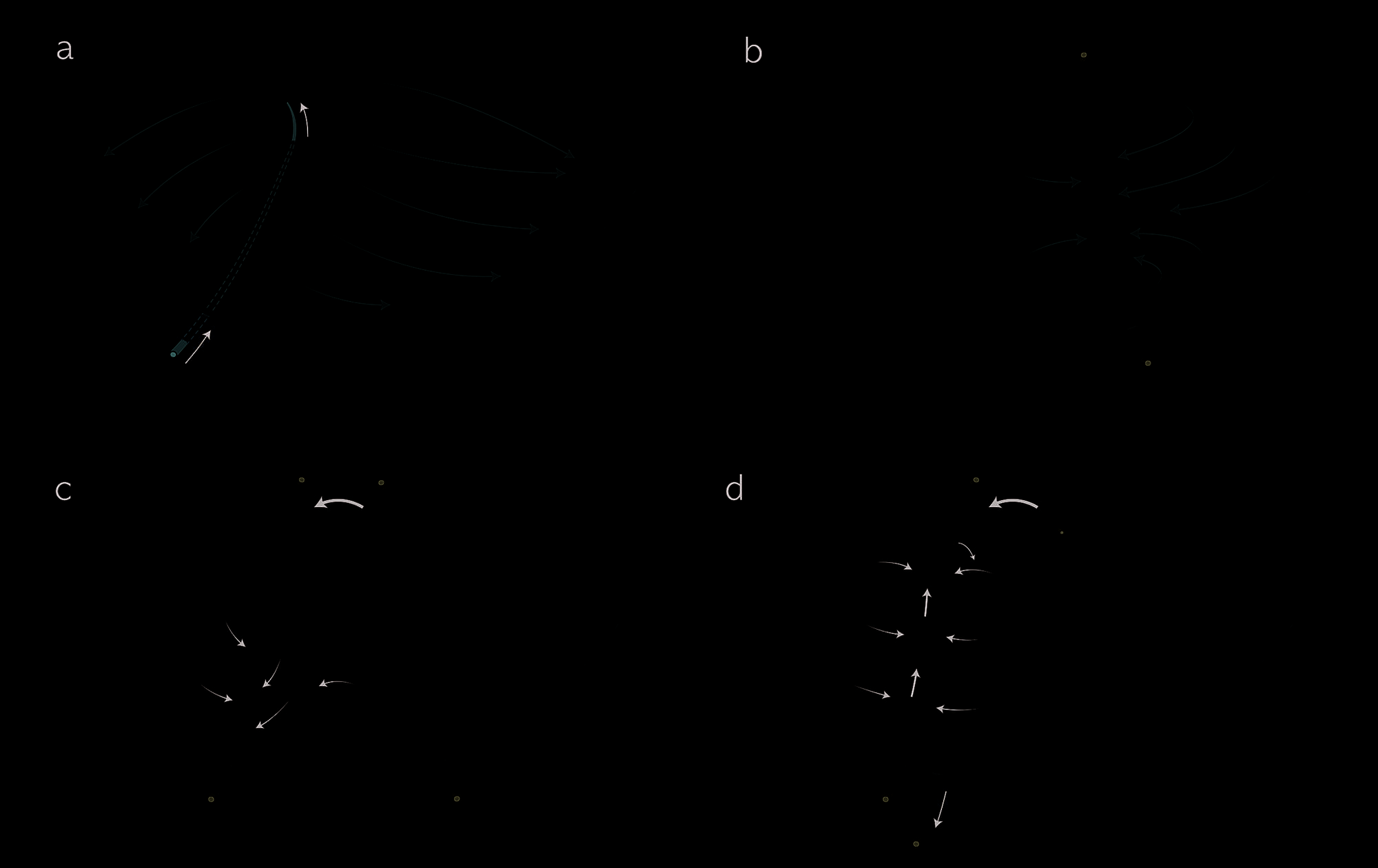

Supplement: S1 File — (ZIP) [file pone.0242214.s001.zip › Figure5.tif]

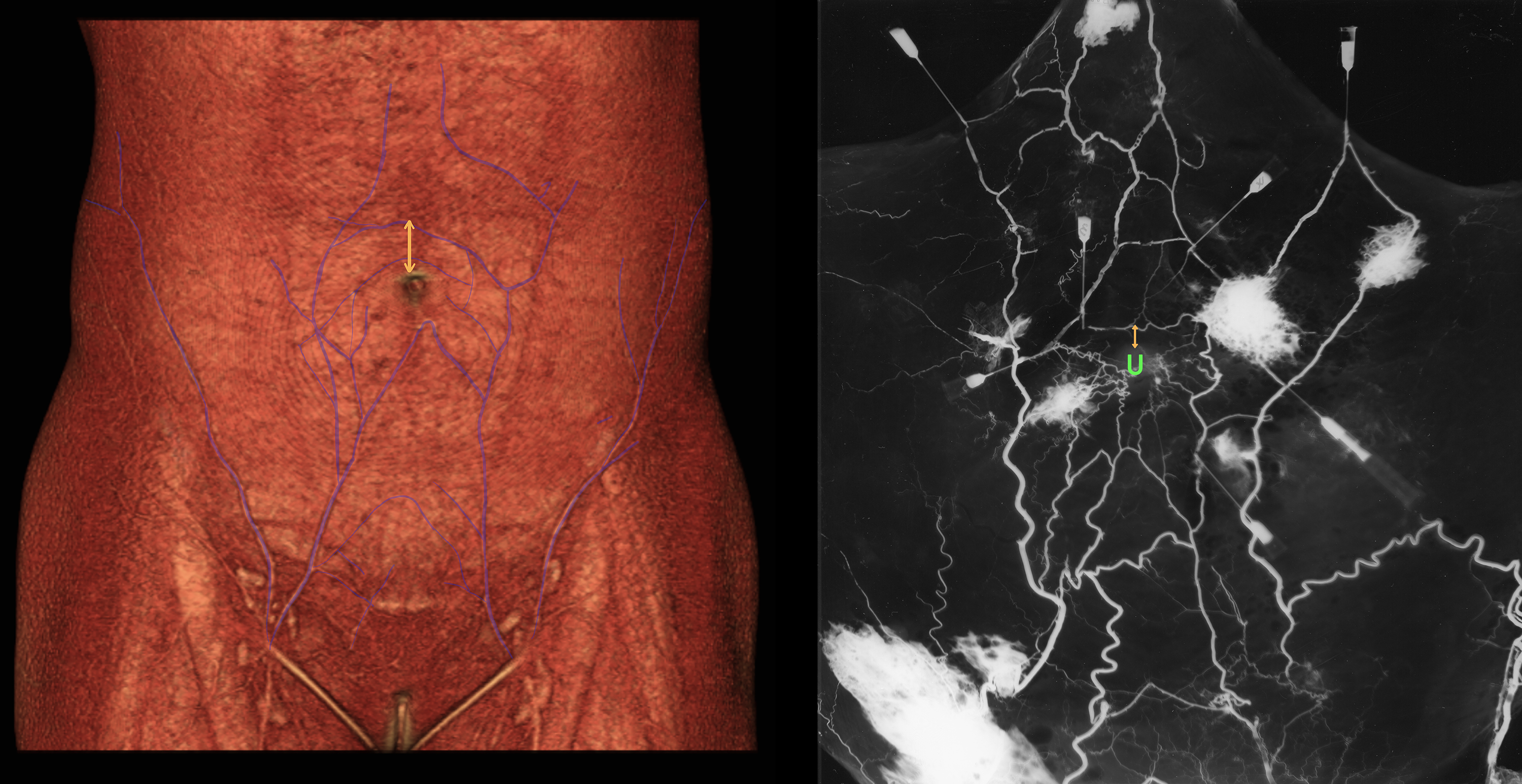

Supplement: S1 File — (ZIP) [file pone.0242214.s001.zip › Figure7.tif]
